# Eucalyptus Essential Oil as a Natural Food Preservative: *In Vivo* and *In Vitro* Antiyeast Potential

**DOI:** 10.1155/2014/969143

**Published:** 2014-08-07

**Authors:** Amit Kumar Tyagi, Danka Bukvicki, Davide Gottardi, Giulia Tabanelli, Chiara Montanari, Anushree Malik, Maria Elisabetta Guerzoni

**Affiliations:** ^1^Applied Microbiology Laboratory, Centre for Rural Development & Technology, Indian Institute of Technology Delhi, New Delhi 110 016, India; ^2^Dipartimento di Scienze degli Alimenti, Università degli Studi di Bologna, Sede di Cesena, Piazza G. Goidanich 60, 47023 Cesena, Italy; ^3^Faculty of Biology, University of Belgrade, Institute of Botany and Botanical Garden “Jevremovac”, 11 000 Belgrade, Serbia; ^4^Interdipartimental Center for Industrial Research-CIRI-AGRIFOOD, Alma Mater Studiorum, University of Bologna, Piazza Goidanich 60, 4752 Cesena, Italy

## Abstract

In this study, the application of eucalyptus essential oil/vapour as beverages preservative is reported. The chemical composition of eucalyptus oil was determined by gas chromatography-mass spectrometry (GC-MS) and solid phase microextraction GC-MS (SPME/GC-MS) analyses. GC-MS revealed that the major constituents were 1,8-cineole (80.5%), limonene (6.5%), *α*-pinene (5%), and *γ*-terpinene (2.9%) while SPME/GC-MS showed a relative reduction of 1,8-cineole (63.9%) and an increase of limonene (13.8%), *α*-pinene (8.87%), and *γ*-terpinene (3.98%). Antimicrobial potential of essential oil was initially determined in vitro against 8 different food spoilage yeasts by disc diffusion, disc volatilization, and microdilution method. The activity of eucalyptus vapours was significantly higher than the eucalyptus oil. Minimum inhibitory concentration (MIC) and minimum fungicidal concentration (MFC) varied from 0.56 to 4.50 mg/mL and from 1.13 to 9 mg/mL, respectively. Subsequently, the combined efficacy of essential oil and thermal treatment were used to evaluate the preservation of a mixed fruit juice in a time-dependent manner. These results suggest eucalyptus oil as a potent inhibitor of food spoilage yeasts not only in vitro but also in a real food system. Currently, this is the first report that uses eucalyptus essential oil for fruit juice preservation against food spoiling yeast.

## 1. Introduction

*Eucalyptus* is an evergreen, tall tree, or shrub, belonging to Myrtaceae family. Although it is native to Australia and Tasmania, nowadays it has extensively spread to other countries [1]. The genus* Eucalyptus* contains about 700 species; among them, more than 300 contain volatile oils in their leaves. Essential oils of various eucalyptus species are used in the pharmaceutical, toiletries, cosmetics, and food industries [1]. These broad applications are due to the antiseptic, antihyperglycemic, anti-inflammatory, flavouring, and antioxidant properties of the molecules present in the oil [2]. The antimicrobial activity of eucalyptus oils has been found to vary significantly within microbial species and within microbial strains. The strong antimicrobial activity may be directly associated with their major compounds in the oil (such as 1,8-cineole and *α*-pinene) or with the synergy between the major and minor constituents [3]. Previous results reported that Gram positive bacteria are more susceptible than Gram negative bacteria; moreover, activity against fungi and yeasts (*Candida albicans* and* Saccharomyces cerevisiae*) has also been detected [4]. According to one of the reports,* Eucalyptus odorata* had the strongest activity against bacteria and yeasts while* E. bicostata* had the best antiviral activity [3]. Although several studies about eucalyptus oils have been published [5–7], only few of them evaluated their activity against pathogenic and food spoilage microorganisms [4, 8]. Despite the well-reported antimicrobial activity* in vitro*, the food industry has applied eucalyptus essential oils mainly as flavouring agents. Therefore, the use of essential oils as preservatives in food has been limited. Because the required concentration against microorganisms is affected by the interactions of the oil compounds with the food matrix components, higher concentrations are needed to achieve sufficient activity. This negatively impacts the organoleptic properties of the final product [9]. To overcome this problem, a promising alternative is the use of a combination of mild temperature treatment with essential oils [10]. A mild thermal treatment, in fact, enhances the antimicrobial efficacy of the essential oil influencing the vapour pressure of the molecules [11].

In the present study, after a chemical characterization by GC-MS,* in vitro* effect of eucalyptus oil against 8 different food spoilage yeast species was studied through the disc diffusion method, the disc volatilisation method, and MIC/MFC. Moreover, to evaluate the antiyeast activity* in vivo*, we employed a real food system based on the preservation of a mixed fruit juice inoculated with* S. cerevisiae* and stored at room temperature for 8 days. Further, in order to improve the efficiency of the essential oil, the combined effect of oil and thermal treatment was also evaluated in the same real system.

## 2. Materials and Methods

### 2.1. Chemicals and Strains

The essential oil was procured from Erbamea, “*olio essenziale naturale,*" Italy, and stored in an airtight sealed glass bottle at 4°C till further use. Growth media and Tween 80 were purchased from Oxoid Ltd., Basingstoke, Hampshire, UK, and Merck Schuchardt, Germany, respectively. Different yeast strains (*Saccharomyces cerevisiae* SPA,* Zygosaccharomyces bailii* 45,* Aureobasidium pullulans* L6F,* Candida diversa* T6D,* Pichia fermentans* T2A1,* Pichia kluyveri* T1A,* Pichia anomala*, and* Hansenula polymorpha* CBS 4732) were obtained from the strain collection of the Dipartimento di Scienze degli Alimenti, University of Bologna, Italy, and used to evaluate the effect of essential oil. The yeast strains were grown in yeast peptone dextrose (YPD) medium at 28°C for 24 h in an orbital shaking incubator (Universal Table Shaker 709, Milan, Italy) at 120 rpm.

### 2.2. Gas Chromatography-Mass Spectrometry (GC-MS) and Solid Phase Microextraction-Gas Chromatography-Mass Spectrometry (SPME/GC-MS) Analyses of Eucalyptus Essential Oil

GC-MS analyses were carried out on an Agilent 7890 gas chromatograph (Agilent Technologies, Palo Alto, CA) coupled to an Agilent 5975 mass selective detector operating in electron impact mode (ionization voltage, 70 eV). A CP-Wax 52 CB capillary column (50 m length, 0.32 mm inner diameter, and 1.2 *μ*m film diameter) was used. The temperature program started from 50°C and then was programmed at 3°C/min to 240°C which was maintained for 1 min. Injector, interface, and ion source temperatures were 250°C, 250°C, and 230°C, respectively. Injections were performed in split mode and helium (1 mL/min) was used as carrier gas. The mass selective detector was operated in the scan mode between 20 and 400 m/z. Data acquisition started 4 min after injection. Five millilitres of 10 ppm solution of the eucalyptus oil was placed in 10 mL vials and the vials were sealed by PTFE/silicone septa. 1 *μ*L of the samples was injected directly into the column with a split ratio of 1 : 100. Component separation was achieved following the method described above.

For the SPME analysis, a divinylbenzene-poly(dimethylsiloxane) coated stable flex fiber (65 *μ*m) and a manual SPME holder (Supelco Inc., Bellefonte, PA, USA) were used in this study after preconditioning according to the manufacturer's instruction manual. Samples were put into sealed vials for 10 min at room temperature. The SPME fiber was exposed to each sample for 10 min by manually penetrating the septum, and, finally, the fiber was inserted into the injection port of the GC for 10 min sample desorption.

The identification of the molecules was based on comparison of mass spectra of compounds both with those contained in available databases (NIST version 2005) and with those of pure standards (Sigma-Aldrich, Milan, Italy) analyzed under the same conditions.

### 2.3. Antiyeast Activity of Eucalyptus Oil and Vapour

#### 2.3.1. Disc Diffusion Method

The agar disc diffusion method was employed for the determination of antimicrobial activities of the essential oils [12] (NCCLS, 1997). Briefly, a suspension of the tested microorganism (100 *μ*L of 1 × 10^6^ CFU/mL) was confirmed by viable counts and spread on the YPD agar media plates. These plates were allowed to dry. Filter paper discs, 6 mm in diameter (Schleicher & Schuell, Germany), were soaked with 10 *μ*L of the oil and placed on the inoculated plates and, after storing at 4°C for 2 h, were incubated at 28°C for 48 h. Volume of essential oils tested was varied (10, 20, or 30 *μ*L) by using appropriate number of sterile discs. The diameters of the inhibition zones were measured in millimetres.

#### 2.3.2. Disc Volatilisation Method

Standard experimental setup as described by López et al. [13] was used. Briefly, a 100 *μ*L portion of each suspension containing approximately 10^6^ CFU/mL was spread over the surface of YPD agar plate and allowed to dry. A paper disc (diameter 6 mm; Schleicher & Schuell, Germany) was laid on the inside surface of the upper lid and 10 *μ*L eucalyptus oil was soaked on each disc. The plates inoculated with microorganisms were immediately inverted on top of the lid and sealed with parafilm to prevent leakage of eucalyptus oil vapour. Plates were incubated at 28°C for 48 h and the diameter of the resulting inhibition zone in the yeast lawn was measured.

#### 2.3.3. Determination of Minimum Inhibitory Concentration (MIC) and Minimum Fungicidal Concentration (MFC)

Broth microdilution assays were performed as recommended by NCCLS [12]. All tests were performed in YPD agar supplemented with Tween 80 (final concentration of 0.5% v/v). Yeast strains were cultured overnight at 28°C in YPD broth. Test strains were suspended in YPD to give a final density of 1 × 10^6^ CFU/mL. Geometric dilutions ranging from 0.036 mg/mL to 72 mg/mL of the eucalyptus oil were prepared in a 96-well microtiter plate, including one growth control (YPD broth + Tween 80) and one sterility control (YPD broth + Tween 80 + test oil). Plates were incubated at 30°C for 48 h. The yeast cell growth was indicated by the presence of a white pellet on the well bottom. The MIC values were determined as the lowest concentration of oil preventing visible growth of microorganisms. MFC was defined as the lowest concentration at which no growth was observed after subculturing into fresh media.

### 2.4. Mixed Fruit Juice Preservation by Eucalyptus Oil and Thermal Treatment

#### 2.4.1. Preparation of Fruit Juice Mixture Inoculated with* S. cerevisiae* SPA

Apples (Golden Delicious) and oranges at commercial maturity were purchased from a local market (Ipercoop, Cesena). After being washed, apples were cut into about 35 × 25 × 5 mm slices and then immersed in 0.2% ascorbic acid solution (to avoid undesirable enzymatic browning during the processing), drained quickly, and made into juices using a blender. After being washed, oranges were peeled off and made into juices. Both juices were mixed in 1 : 1 ratio. The suspension of yeast strain (*S. cerevisiae* SPA) was mixed with fruit juice mixture to result in final concentration of 10^3^ CFU/mL and the inoculated juice mixtures were transferred into 10 mL sterilized glass vials.

#### 2.4.2. Effect of Thermal Treatment

The effect of thermal treatment was studied by exposing the mixed juice samples at 70°C for 30, 60, and 90 s. Subsequently, the treated vials were stored at room temperature up to 8 days and samples were drawn on 0, 2nd, 4th, and 8th day.

#### 2.4.3. Effect of Eucalyptus Oil

1.0% ethyl alcohol solution of eucalyptus oil was mixed in the inoculated fruit juice mixture at MIC level (4.5 mg/mL), half of MIC level (2.25 mg/mL), and one-fourth of MIC level (1.125 mg/mL). Fruit juice sample inoculated with* S. cerevisiae* alone was considered as positive control. Subsequently, the treated vials were stored at room temperature up to 8 days and samples were drawn on 0, 2nd, 4th, and 8th day.

#### 2.4.4. Effect of Eucalyptus Oil and Thermal Treatment: Combined Effect

A set of inoculated fruit juice mixtures vials added with three different concentrations of eucalyptus oil were exposed to thermal treatment (70°C) for 30, 60, and 90 s. Each condition was treated in triplicate. Subsequently, the treated vials were stored at room temperature up to 8 days and samples were drawn on 0, 2nd, 4th, and 8th day. All treated samples were serially diluted and plated on PDA. The plates were incubated for 72 h at 28°C and CFU counts were made. The efficacy of the thermal treatment alone and the combination with different doses of eucalyptus oil were quantified in time-dependent manner by the variation in log CFU/mL of the inoculated yeast strains.

### 2.5. Statistical Analyses

All the experiments were done in triplicate and repeatability was established. Significance of differences among treatments (*P* ≤ 0.05) was analysed using one-way ANOVA (SPSS, 10.0 version). For all experiments, three replicates were used and the data presented here represents the mean of these replicates with standard error or deviation. Moreover, as regards yeast load counts during juice storage, a principal component analysis (PCA) was carried out with Statistica 6.1 (StatSoft Italy srl, Vigonza, Italy), using the different concentrations of eucalyptus essential oil and duration of the thermal treatments as variables.

## 3. Results and Discussion

### 3.1. Chemical Composition of Eucalyptus Oil

Thirteen compounds were identified by GC-MS for the total of 99.7% (Table 1). The major constituents of the oil were 1,8-cineole (80.4%), followed by limonene (6.5%), *α*-pinene (5%), and *γ*-terpinene (2.9%). On the contrary, seventeen molecules were detected by SPME/GC-MS for the total of 99.9% (Table 2). The major constituents were the same as the reported ones in Table 1 but with different relative composition: 1,8-cineole (63.9%), limonene (13.8%), *α*-pinene (8.9%), and *γ*-terpinene (3.9%). The differences between the chemical contents of oil and vapour and their reasons were also evaluated in our previous reports [4]. As reported in the literature, essential oil of eucalyptus was characterized by very high concentration of 1,8-cineole. Damjanović-Vratnica et al. [14] determined an 85.8% 1,8-cineole in eucalyptus essential oil from Montenegro and reported its significant activity against different bacteria and yeasts. Moreover, Elaissi et al. [3] showed strong antibacterial, antifungal, and antiviral effect of eight eucalyptus oils from Tunisia.

### 3.2. Antiyeast Activity of Eucalyptus Oil

In this work, the antiyeast activity of eucalyptus oil was evaluated with 8 different food spoilage yeasts:* S. cerevisiae*,* Z. bailii*,* A. pullulans*,* C. diversa*,* P. fermentans*,* P. kluyveri*,* P. anomala*, and* H. polymorpha.*

#### 3.2.1. Disc Diffusion Method

**Figure 1:**
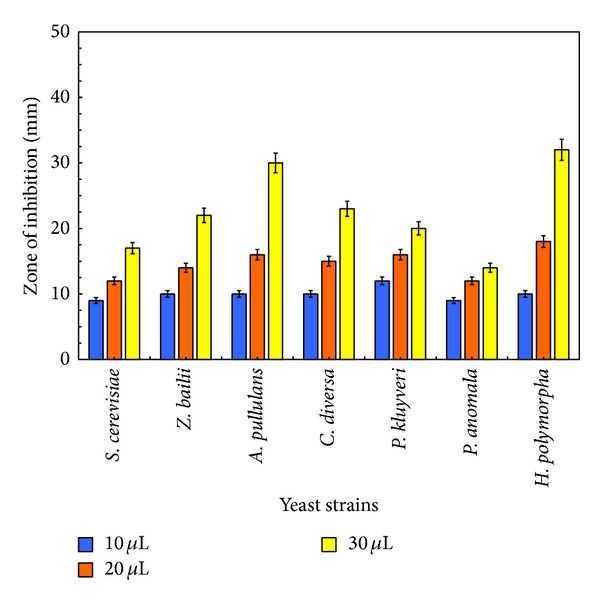
Antiyeast potential of eucalyptus oil evaluated with disc diffusion method. Zone of inhibition due to the different concentrations (10, 20, and 30 *μ*L) of eucalyptus oil against* S. cerevisiae* SPA,* Z. bailii* 45,* A. pullulans* L6F,* C. diversa* T6D,* P. kluyveri* T1A,* P. anomala*, and* H. polymorpha* CBS 4732 was measured. Column height represents the mean of triplicate results and error bar represents the standard deviation.

The antiyeast activity of eucalyptus essential oil was assessed by the presence or the absence of inhibition zones. Three different concentrations of the oil (10, 20, and 30 *μ*L) were tested. The highest susceptible yeast was* H. polymorpha* (10, 18, and 32 mm), followed by* A. pullulans* (10, 16, and 30 mm),* C. diversa* (10, 15, and 23 mm),* Z. bailii* (10, 14, and 22 mm),* P. kluyveri* (12, 16, and 20 mm), and* S. cerevisiae* (9, 12, and 17 mm) (Figure 1).* P. anomala* presented the lowest inhibition zone (9, 12, and 14 mm). The results demonstrated that eucalyptus oil was effective against all the considered strains. Previous data already reported that eucalyptus oil possesses antimicrobial activity against different microorganisms [14–18]. For example,* Eucalyptus staigeriana* oil showed strong activity (with 14.3–18.2 mm zones of inhibition) against several microorganisms (*Escherichia coli*,* Staphylococcus aureus*, and* Alcaligenes faecalis*) and no activity against yeast* C*.* albicans* [19].* Eucalyptus cinerea* oil exhibited significant activity against* S. pyogenes* (26 mm) and* P. aeruginosa* (17 mm). The zone of inhibition for* S. aureus* was 13 mm. Only isolated and purified 1,8-cineole (eucalyptol) presented no antimicrobial activity against* S. aureus* and* C. albicans* [20]. Also, Vilela et al. [21] tested the antimicrobial activity of both the eucalyptus essential oil and 1,8-cineole against two* Aspergillus* species. They reported a complete fungal growth inhibition when using the essential oil, while a reduced activity was detected when using 1,8-cineole alone. This shows that possible synergistic effect of minor and major components determines the final antimicrobial activity of the essential oils [22]. Based on the chemical composition, it can be concluded that the antimicrobial activity of the oil is apparently attributed to its high content of oxygenated monoterpenes.

#### 3.2.2. Disc Volatilisation Method

**Figure 2:**
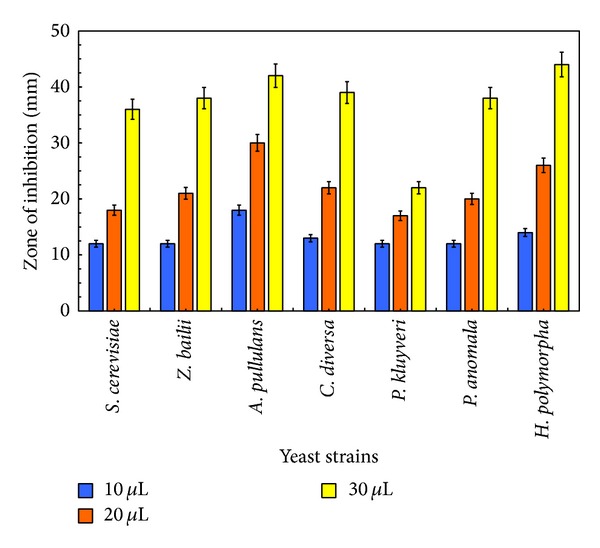
Antiyeast potential of eucalyptus oil vapour evaluated with disc volatilisation method. Zone of inhibition due to the different concentrations (10, 20, and 30 *μ*L) of eucalyptus oil against* S. cerevisiae* SPA,* Z. bailii* 45,* A. pullulans* L6F,* C. diversa* T6D,* P. kluyveri* T1A,* P. anomala*, and* H. polymorpha* CBS 4732 was measured. Column height represents the mean of triplicate results and error bar represents the standard deviation.

The zone of inhibition in yeast strains due to eucalyptus essential oil vapours is shown in Figure 2. Zone of inhibition due to essential oil vapour increased in a dose-dependent manner similar to disc diffusion method. The inhibition zones observed using 30 *μ*L of eucalyptus oil vapours were* P. kluyveri* (22 mm) <* S. cerevisiae* (36 mm) <* Z. bailii* (38 mm) =* P. anomala* (38 mm) <* C. diversa* (39 mm) <* A. pullulans* (42 mm) <* H. polymorpha* (44 mm).* H. polymorpha* was the most susceptible yeast to eucalyptus oil vapours since 14, 26, and 44 mm inhibition zones were generated using 10, 20, and 30 *μ*L eucalyptus oil vapours, respectively (Figure 2). As compared to the oil, the eucalyptus vapours resulted in a significantly larger zone of inhibition (*P* ≤ 0.05) in all the strains tested. This better result could be attributed to the variation in relative composition of the oil and vapours, as already shown in Table 2.

#### 3.2.3. MIC and MFC of Eucalyptus Oil

MIC and MFC of eucalyptus essential oil were determined against food spoiling yeasts (Table 3). The MIC values varied from 0.56 mg/mL to 4.5 mg/mL. MIC value for* S. cerevisiae* and* A. pullulans* was higher (i.e., 4.5 mg/mL) than that for* Z. bailii*,* C. diversa*,* P. fermentans*, and* H. polymorpha* (i.e., 2.5 mg/mL).* P. anomala* and* P. kluyveri* showed the lowest MIC values, 1.13 mg/mL and 0.56 mg/mL, respectively. MFC varied from 1.13 mg/mL to 9 mg/mL and showed a similar pattern to MIC; that is,* S. cerevisiae* and* A. pullulans* (9 mg/mL) had higher values than* Z. bailii*,* C. diversa*,* P. fermentans*,* H. polymorpha* (4.5 mg/mL),* P. anomala.* (2.25 mg/mL), and* P. kluyveri* (1.13 mg/mL). Silva et al. [20] reported the minimal inhibitory concentrations of the eucalyptus essential against different bacteria:* Streptococcus pyogenes* (MIC: 0.39 mg/mL),* Staphylococcus aureus* (MIC: 0.78 mg/mL),* Pseudomonas aeruginosa* (MIC: 1.56 mg/mL), and* Candida albicans* (MIC: 0.78 mg/mL). Damjanović-Vratnica et al. [14] reported that the MICs of eucalyptus oil from Montenegro against 17 microorganisms, including food poisoning and spoilage bacteria and human pathogens, varied between 0.3 and 3.13 mg/mL, which can be attributed to the different amount of active molecules observed in the tested eucalyptus oils. In fact, according to Soković et al. [23] and Inouye et al. [24], not only the major compounds (1,8-cineole) but also the minor ones (such as *γ*-terpinene, *α*-pinene, *β*-pinene, myrcene, and linalool) play a significant role in the antimicrobial activity.

### 3.3. Mixed Fruit Juice Preservation by Eucalyptus Oil and Thermal Treatment

#### 3.3.1. Effect of Thermal Treatment

**Figure 3:**
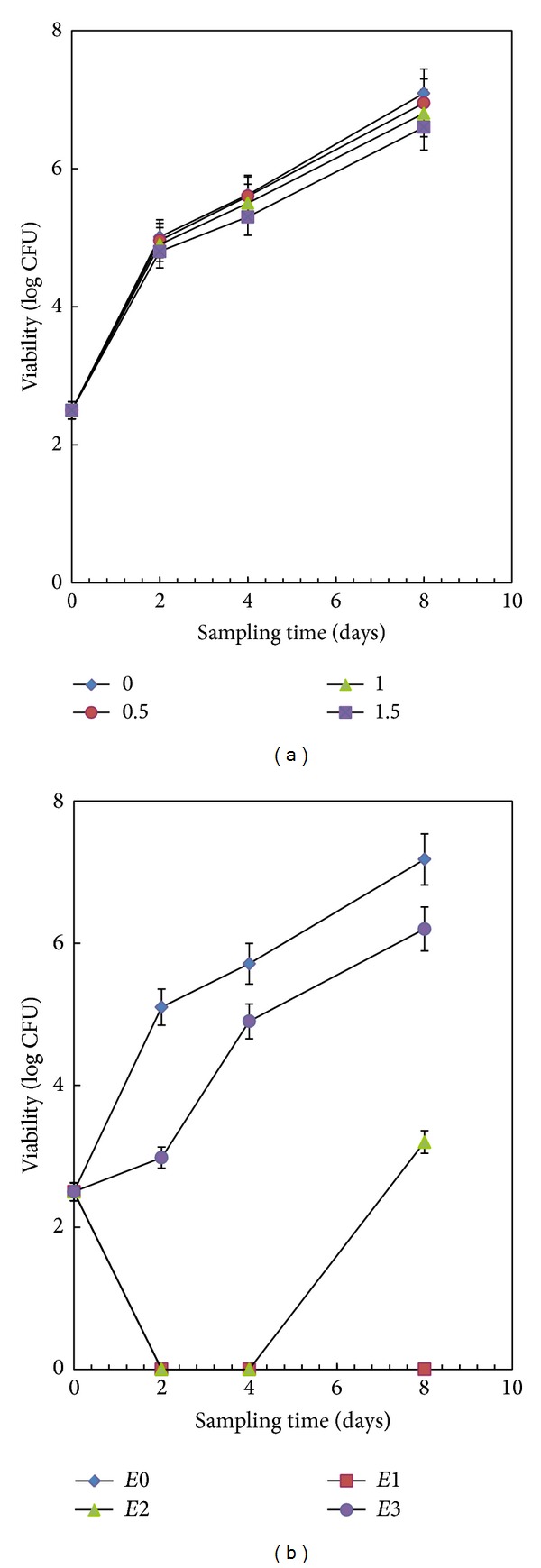
Variation in viability of* S. cerevisiae* SPA in fruit juice mixtures during storage after (a) thermal treatment at 70°C for 0.5, 1, and 1.5 min; (b) eucalyptus oil treatment at different concentrations (*E*1 = MIC, *E*2 = 1/2 MIC, *E*3 = 1/4 MIC level, and *E*0 = control). The data represents the mean of triplicate results and error bar represents standard deviation.

A thermal treatment for 30 and 60 s at 70°C did not have effect on the growth of* S. cerevisiae* in the mixed fruit juice samples (Figure 3(a)). Indeed, only a 0.49 log CFU/mL reduction was observed in samples, subjected to a thermal treatment for 90 sec, after eight days at room temperature. Hence, this kind of treatment was almost ineffective for preserving the juice. A similar pattern was also observed in our previous studies [25, 26].

#### 3.3.2. Effect of Varying Concentrations of Eucalyptus Oil

Since eucalyptus oil was able to kill several food spoilage yeasts in* in vitro* tests, its activity in a real food system (mixed fruit juice) has also been studied. The reduction inviability of* S. cerevisiae* due to eucalyptus oil treatment in dose-dependent manner (MIC, 1/2 MIC, and 1/4 MIC level) and time-dependent manner (i.e., 0, 2, 4, and 8 days) was evaluated. As shown in Figure 3(b), a complete growth inhibition was observed in the mixed fruit juice when MIC levels of essential oil were used. However, 1/2 MIC and 1/4 MIC level samples showed a significant reduction in the final number of cells (3.2 log CFU/mL and 6.2 log CFU/mL, resp.) compared to untreated sample (7.2 log CFU/mL). These data represented that the yeast growth has been inhibited in a dose-dependent manner even in food matrix. As previously reported [27], different essential oils showed an excellent activity against food spoilage yeasts (*Saccharomyces cerevisiae*,* Candida rugosa*,* Debaryomyces hansenii*,* Kluyveromyces marxianus*,* Rhodotorula glutinis*,* Rhodotorula minuta*,* Trichosporon cutaneum*,* Yarrowia lipolytica,* and* Zygosaccharomyces rouxii*). For example, cardamom oil acted as a good antimicrobial agent in real system such as pine apple fruit juice [28], sweet orange juice [29], and apple juice [30]. In the present study, it is the first attempt to evaluate the antiyeast potential of eucalyptus oil in fruit juice mixture. In some cases, the natural compounds performed even better than the chemical preservatives [27].

#### 3.3.3. Combined Effect of Eucalyptus Oil and Thermal Treatment

**Figure 4:**
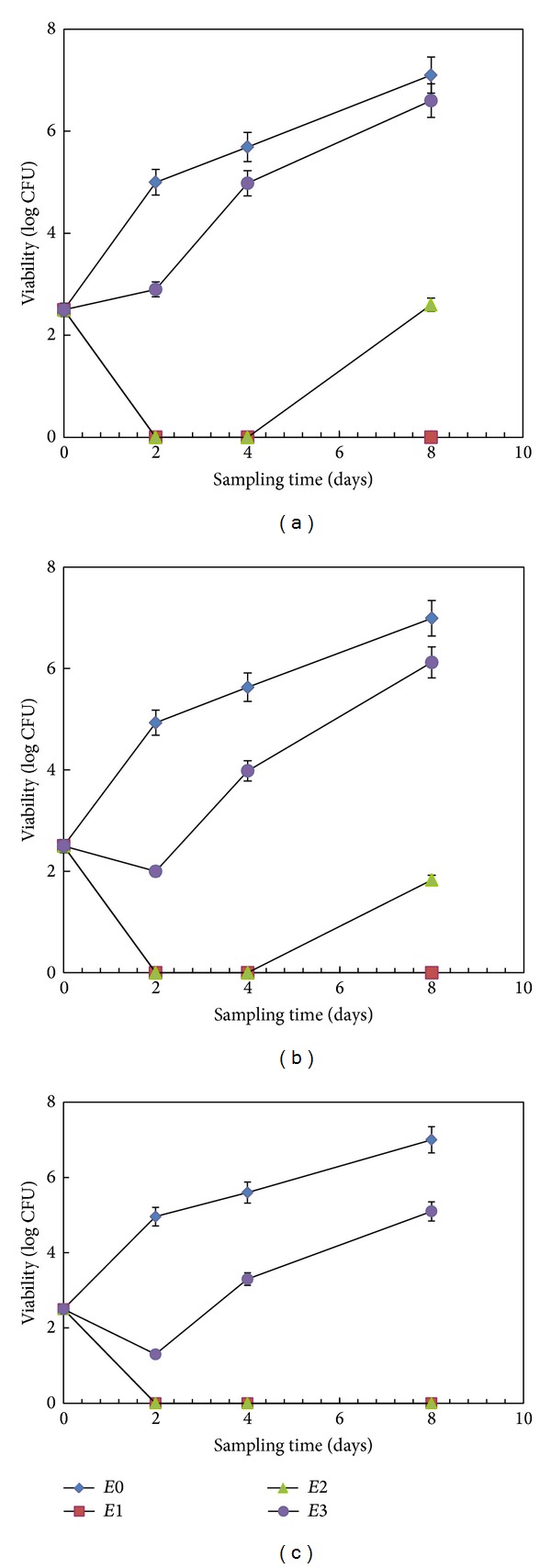
Combined effect of essential oil and thermal treatment. Variation in viability of* S. cerevisiae* SPA in fruit juice mixtures was estimated. Different concentrations of eucalyptus oil (*E*1 = MIC, *E*2 = 1/2 MIC, *E*3 = 1/4 MIC level, and *E*0 = control) combined with different thermal treatments at 70°C for (a) 30, (b) 60, and (c) 90 sec were tested. The growth of the yeast was followed up to 8 days after the treatment. The data represents the mean of triplicate results and error bar represents standard deviation.

The combined effect of eucalyptus oil (at MIC, 1/2 MIC, and 1/4 MIC level) with thermal treatment (at 70°C for 30, 60, and 90 sec) on* S. cerevisiae* growth was determined in a time-dependent manner (i.e., 0, 2, 4, and 8 days) at room temperature (Figure 4). MIC and 1/2 MIC levels of eucalyptus oil combined with thermal treatment showed complete growth inhibition of* S. cerevisiae* after two days. In fact, the same growth recovery was also found in samples treated with only eucalyptus oil at 1/2 MIC dose up to eight days of storage. However, the 30 and 60 sec thermal treatments combined with the oil produced a final reduction of 4.5 and 5.16 log CFU/mL, respectively (Figure 4), when compared with 3.98 log CFU/mL measured in those treated with essential oil alone (Figure 3(b)). Finally, the samples with 1/4 MIC level of the oil were not affected by 30 sec thermal treatment, if compared with Figure 3(b). Nevertheless, the 60 and 90 sec of thermal treatments reduced the growth by 0.89 to 1.90 log CFU/mL, respectively, as compared to the control.

It has been reported that the use of thermal treatment affects the volatile compounds by increasing their vapour pressure, which in turn improves the possibility to solubilize the yeast cell membrane. Though, the use of only one treatment cannot guarantee the microbial stability of the beverages without affecting the final organoleptic properties of the product [31]. The combination of thermal treatment with essential oils offers a very useful synergy whereby increase in temperatures during storage could enhance the vapour phase concentration of volatiles, thereby enhancing the antimicrobial activity for better food preservation [25]. In some of our previous reports [32–34], it was also observed that antimicrobial activity of essential oils was higher in vapour phase than in liquid phase, which was observed by different microscopic techniques: scanning electron microscope, transmission electron microscope, and atomic force microscope. Basically, the differences in inhibition of yeast strain obtained from essential oil (liquid phase, direct contact with the culture media) and the vapour can be attributed to the differences in diffusion coefficients of the antimicrobial compounds present in the eucalyptus oil when they have to diffuse in the agar compared to the diffusion in vapour phase [35]. In our study, the oil dose requirement was significantly reduced with the combination of the two treatments. This combination can be used as a better preservative with minimal impact on the organoleptic properties of the beverage. Even our previous studies using a combination of oils (mentha and lemongrass) and thermal treatment have been published [25, 26]; this is the first report that uses eucalyptus essential oil for fruit juice preservation against* S. cerevisiae*.

#### 3.3.4. Principal Component Analysis

**Figure 5:**
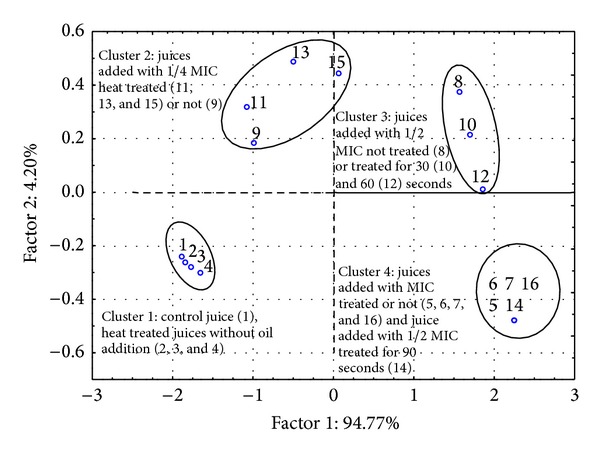
PCA loadings plot on the two first factors of control and treated juice. The clusters generated were as follows. Cluster 1: control juice (1), heat treated juices without essential oil (2, 3, and 4). Cluster 2: juices added with 1/4 MIC heat treated (11, 13, and 15) or not (9). Cluster 3: juices added with 1/2 MIC not treated (8) or treated for 30 (10) and 60 (12) seconds. Cluster 4: juices added with MIC treated or not (5, 6, 7, and 16) and juice added with 1/2 MIC treated for 90 seconds (14).

In order to confirm the interactive effects between the different variables (concentrations of eucalyptus essential oil and thermal treatments) on the yeast growth, a principal component analysis (PCA) was carried out.  Figure 5 reports the PCA loadings plot on the first two factors of the samples. As expected, factor 1 (essential oil concentration) accounted for the great part of variability (about 94%) while factor 2 (thermal treatment) had a limited effect. In particular, four clusters can be identified. In the first, the control juice and the heat treated juices (without eucalyptus essential oil) were grouped. In the second, the juices added with 1/4 MIC thermal treated or not were grouped together. This cluster was characterized by a lower difference compared with cluster 1 in relation to factor 1. This means that the addition of this amount of oil had scarce activity on the effectiveness of heat treatment on yeast viability during storage. At last, clusters 3 and 4 were characterized by pronounced differences with respect to the sample without essential oil. Cluster 3 was formed by juices with 1/2 MIC, not treated and thermal treated for 30 and 60 seconds. The last cluster grouped all the samples having better antiyeast results (juices with MIC level and the sample with 1/2 MIC thermal treated for 90 seconds). The composition of the latter cluster highlights the equivalence of the antiyeast effectiveness of this last sample with the juices containing a double concentration of oil. This fact can allow for obtaining the same antiyeast effect using a concentration of essential oil with a lower impact on organoleptic profile of juices.

## 4. Conclusion

The results of this work demonstrated that eucalyptus essential oil could be used as a potential antimicrobial compound against food spoilage yeasts (*in vitro* and in a real food system). The chemical identification of the different molecules characterizing the eucalyptus oil evidenced the presence of oxygenated monoterpenes responsible for the antimicrobial activity. The use of the combination of eucalyptus essential oil with thermal treatment successfully inhibited the development of yeast (*S. cerevisiae* SPA) in fresh fruit juices. The results provide an excellent record of eucalyptus oil as antimicrobial agent and suggest its potential application for beverages preservation. Additional studies should be conducted to confirm the potentiality of eucalyptus essential oil in order to use it as a preservative in other food models.

## Figures and Tables

**Table 1:** Chemical constituents of eucalyptus essential oil obtained through GC-MS.

RT (min)	Compound	Percentage
15.776	*α*-Pinene	5.02
17.843	*β*-Pinene	0.54
18.267	*β*-Myrcene	0.77
19.092	*α*-Phellandrene	0.53
19.685	Terpinolene	0.10
20.128	Limonene	6.45
20.646	1,8-Cineole	80.44
21.75	*γ*-Terpinene	2.90
23.237	4-Carene	0.34
23.616	Linalool	0.16
25.889	Pinocarveol	0.17
27.69	4-Terpineol	0.55
28.296	*α*-Terpineol	1.72
	Monoterpene hydrocarbons	16.67
	Oxygenated monoterpenes	83.04
	Total of identified compound	99.71

RT: retention time (min), relative area percentage. Results are based on GC-MS. MS acquisition started after 4 min.

**Table 2:** Chemical constituents of eucalyptus essential oil obtained through SPME/GC-MS.

RT (min)	Compound	Percentage
8.069	*α*-Pinene	8.87
11.315	*β*-Pinene	0.82
13.422	*β*-Myrcene	1.07
13.677	*α*-Phellandrene	1.05
13.908	3-Carene	0.19
14.323	Terpinolene	0.19
15.221	Limonene	13.84
15.877	1,8-Cineole	63.96
17.255	*γ*-Terpinen	3.98
18.423	o-Cymene	4.13
18.948	4-Carene	0.15
22.443	*trans*-5-Methyl-2-isopropyl-2-hexen-1-al	0.05
25.958	p-Cymene	0.14
30.132	Linalool	0.10
32.868	4-Terpineol	0.26
35.08	Pinocarveol	0.08
36.609	*α*-Terpineol	0.43
	Monoterpene hydrocarbons	34.46
	Oxygenated monoterpenes	64.83
	Total of identified compound	99.29

RT: retention time (min), relative area percentage. Results are based on GC-MS.

**Table 3:** MIC/MFC of eucalyptus oil for different yeast strains.

S. number	Name of the strain	MIC (mg/mL)	MFC (mg/mL)
1	*Saccharomyces cerevisiae* SPA	4.5	9
2	*Zygosaccharomyces bailii* 45	2.25	4.5
3	*Aureobasidium pullulans* L6F	4.5	9
4	*Candida diversa* T6D	2.25	4.5
5	*Pichia fermentans* T2A1	2.25	4.5
6	*Pichia kluyveri* T1A	0.56	2.25
7	*Pichia anomala *	1.13	2.25
8	*Hansenula polymorpha* CBS 4732	2.25	4.5

MIC: minimum inhibitory concentration by microbroth dilution method; MFC: minimum fungicidal concentration by streak plate method (*n* = 3; *P* ≤ 0.05).

## References

[1] Marzoug HNB, Romdhane M, Lebrihi A (2011). *Eucalyptus oleosa* essential oils: chemical composition and antimicrobial and antioxidant activities of the oils from different plant parts (stems, leaves, flowers and fruits). *Molecules*.

[2] Takahashi T, Kokubo R, Sakaino M (2004). Antimicrobial activities of eucalyptus leaf extracts and flavonoids from Eucalyptus maculata. *Letters in Applied Microbiology*.

[3] Elaissi A, Rouis Z, Ben Salem NA (2012). Chemical composition of 8 *eucalyptus* species' essential oils and the evaluation of their antibacterial, antifungal and antiviral activities. *BMC Complementary and Alternative Medicine*.

[4] Tyagi AK, Malik A (2011). Antimicrobial potential and chemical composition of *Eucalyptus globulus* oil in liquid and vapour phase against food spoilage microorganisms. *Food Chemistry*.

[5] Pereira V, Dias C, Vasconcelos MC, Rosa E, Saavedra MJ (2014). Antibacterial activity and synergistic effects between *Eucalyptus globulus* leaf residues (essential oils and extracts) and antibiotics against several isolates of respiratory tract infections (*Pseudomonas aeruginosa*). *Industrial Crops and Products*.

[6] Akthar MS, Degaga B, Azam T (2014). Antimicrobial activity of essential oils extracted from medicinal plants against the pathogenic microorganisms: a review. *Issues in Biological Sciences and Pharmaceutical Research*.

[7] Somda I, Leth V, Sérémé P (2007). Antifungal effect of *Cymbopogon citratus*, *Eucalyptus camaldulensis* and *Azadirachta indica* oil extracts on sorghum feed-borne fungi. *Asian Journal of Plant Sciences*.

[8] Sartorelli P, Marquioreto AD, Amaral-Baroli A, Lima MEL, Moreno PRH (2007). Chemical composition and antimicrobial activity of the essential oils from two species of Eucalyptus. *Phytotherapy Research*.

[9] Hyldgaard M, Mygind T, Meyer RL (2012). Essential oils in food preservation: mode of action, synergies, and interactions with food matrix components. *Frontiers in Microbiology*.

[10] Gardini F, Lanciotti R, Guerzoni ME (2001). Effect of trans-2-hexenal on the growth of *Aspergillus flavus* in relation to its concentration, temperature and water activity. *Letters in Applied Microbiology*.

[11] Belletti N, Kamdem SS, Patrignani F, Lanciotti R, Covelli A, Gardini F (2007). Antimicrobial activity of aroma compounds against *Saccharomyces cerevisiae* and improvement of microbiological stability of soft drinks as assessed by logistic regression. *Applied and Environmental Microbiology*.

[12] National Committee for Clinical Laboratory Standards (NCCLS) (1997). Publication M27-A: reference method for Broth dilution antifungal susceptibility testing of yeasts. *Approved Standard*.

[13] López P, Sánchez C, Batlle R, Nerín C (2005). Solid and vapor-phase antimicrobial activities of six essential oils: susceptibility of selected foodborne bacterial and fungal strains. *Journal of Agricultural and Food Chemistry*.

[14] Damjanović-Vratnica B, Đakov T, Šuković D, Damjanović J (2011). Antimicrobial effect of essential oil isolated from *Eucalyptus globulus* Labill. from Montenegro. *Czech Journal of Food Sciences*.

[15] Bendaoud H, Bouajila J, Rhouma A, Savagnacd A, Romdhanea M (2009). GC/MS analysis and antimicrobial and antioxidant activities of essential oil of eucalyptus radiata. *Journal of the Science of Food and Agriculture*.

[16] Bachir RG, Benali M (2012). Antibacterial activity of the essential oils from the leaves of *Eucalyptus globulus* against *Escherichia coli* and *Staphylococcus aureus*. *Asian Pacific Journal of Tropical Biomedicine*.

[17] Ait-Ouazzou A, Lorán S, Bakkali M (2011). Chemical composition and antimicrobial activity of essential oils of *Thymus algeriensis*, *Eucalyptus globulus* and *Rosmarinus officinalis* from Morocco. *Journal of the Science of Food and Agriculture*.

[18] Elaissi A, Salah KH, Mabrouk S, Larbi KM, Chemli R, Harzallah-Skhiri F (2011). Antibacterial activity and chemical composition of 20 Eucalyptus species' essential oils. *Food Chemistry*.

[19] Wilkinson JM, Cavanagh HMA (2005). Antibacterial activity of essential oils from Australian native plants. *Phytotherapy Research*.

[20] Silva SM, Abe SY, Murakami FS, Frensch G, Marques FA, Nakashima T (2011). Essential oils from different plant parts of *Eucalyptus cinerea* F. Muell. ex Benth. (Myrtaceae) as a source of 1,8-cineole and their bioactivities. *Pharmaceuticals*.

[21] Vilela GR, de Almeida GS, D'Arce MABR (2009). Activity of essential oil and its major compound, 1,8-cineole, from *Eucalyptus globulus* Labill., against the storage fungi *Aspergillus flavus* Link and *Aspergillus parasiticus* Speare. *Journal of Stored Products Research*.

[22] Burt S (2004). Essential oils: their antibacterial properties and potential applications in foods—a review. *International Journal of Food Microbiology*.

[23] Soković MD, Vukojević J, Marin PD, Brkić DD, Vajs V, Van Griensven LJLD (2009). Chemical composition of essential oils of Thymus and mentha species and their antifungal activities. *Molecules*.

[24] Inouye S, Takizawa T, Yamaguchi H (2001). Antibacterial activity of essential oils and their major constituents against respiratory tract pathogens by gaseous contact. *Journal of Antimicrobial Chemotherapy*.

[25] Tyagi AK, Gottardi D, Malik A, Guerzoni ME (2013). Anti-yeast activity of mentha oil and vapours through *in vitro* and *in vivo* (real fruit juices) assays. *Food Chemistry*.

[26] Tyagi AK, Gottardi D, Malik A, Guerzoni ME (2014). Chemical composition, *in vitro* anti-yeast activity and fruit juice preservation potential of lemon grass oil. *LWT—Food Science and Technology*.

[27] Kunicka-Styczyńska A (2011). Activity of essential oils against food-spoiling yeast. A review. *Flavour and Fragrance Journal*.

[28] Kapoor IPS, Singh B, Singh G (2008). Essential oil and oleoresins of *Cinnamomum tamala* (tejpat) as natural food preservatives for pineapple fruit juice. *Journal of Food Processing and Preservation*.

[29] Kapoor IPS, Singh B, Singh G (2011). Essential oil and oleoresins of cardamom (*Amomum subulatum* Roxb.) as natural food preservatives for sweet orange (Citrus sinensis) juice. *Journal of Food Process Engineering*.

[30] Friedman M, Henika PR, Levin CE, Mandrell RE (2004). Antibacterial activities of plant essential oils and their components against *Escherichia coli O157:H7* and *Salmonella enterica* in apple juice. *Journal of Agricultural and Food Chemistry*.

[31] Belletti N, Kamdem SS, Tabanelli G, Lanciotti R, Gardini F (2010). Modeling of combined effects of citral, linalool and *β*-pinene used against Saccharomyces cerevisiae in citrus-based beverages subjected to a mild heat treatment. *International Journal of Food Microbiology*.

[32] Tyagi AK, Malik A (2011). Antimicrobial potential and chemical composition of *Mentha piperita* oil in liquid and vapour phase against food spoiling microorganisms. *Food Control*.

[33] Tyagi AK, Malik A (2010). In situ SEM, TEM and AFM studies of the antimicrobial activity of lemon grass oil in liquid and vapour phase against *Candida albicans*. *Micron*.

[34] Tyagi AK, Malik A (2010). Liquid and vapour-phase antifungal activities of selected essential oils against *Candida albicans*: microscopic observations and chemical characterization of *Cymbopogon citratus*. *BMC Complementary and Alternative Medicine*.

[35] Gutiérrez L, Batlle R, Sánchez C, Nerín C (2010). New approach to study the mechanism of antimicrobial protection of an active packaging. *Foodborne Pathogens and Disease*.

